# Release mechanism and parameter estimation in drug-eluting stent systems: analytical solutions of drug release and tissue transport

**DOI:** 10.1093/imammb/dqt025

**Published:** 2014-01-24

**Authors:** Sean McGinty, Sean McKee, Christopher McCormick, Marcus Wheel

**Affiliations:** Department of Mathematics and Statistics, University of Strathclyde, Richmond Street, Glasgow G1 1XH, UK; Biomedical Engineering and Strathclyde Institute of Pharmacy and Biomedical Sciences, University of Strathclyde, Glasgow, UK; Department of Mechanical and Aerospace Engineering, University of Strathclyde, Glasgow, UK

**Keywords:** drug-eluting stents, analytical solutions, parameter estimation, inverse problem

## Abstract

Drug-eluting stents have significantly improved the treatment of coronary artery disease. They offer reduced rates of restenosis compared with their bare-metal predecessors and are the current gold standard in percutaneous coronary interventions. Drug-eluting stents have been approved for use in humans since 2002 and yet, despite the intensive research activity over the past decade, the drug release mechanism(s) and the uptake into the arterial wall are still poorly understood. While stent manufacturers have focussed primarily on empirical methods, several mathematical models have appeared in the literature considering the release problem, the uptake problem and also the coupled problem. However, two significant challenges that remain are in understanding the drug release mechanism(s) and also the determination of the various parameters characterizing the system. These include drug diffusion coefficients and dissolution constants in the stent polymer coating as well as drug diffusion coefficients, binding/uptake rates and the magnitude of the transmural convection in the arterial wall. In this paper we attempt to address these problems. We provide analytical solutions which, when compared with appropriate experiments, may allow the various parameters of the system to be estimated via the inverse problem. The analytical solutions which we provide here for drug release *in vitro* may thus be used as a tool for providing insights into the mechanism(s) of release.

## 1. Introduction

An arterial stent is a small scaffold-like medical device used to increase the size of the lumen when it has become narrowed due to the formation of atherosclerotic plaque. These stents are now routinely coated with a drug to counteract the inflammatory response following insertion into the diseased artery. Compared with their bare metal predecessors, the so-called drug-eluting stents (DESs) offer reduced rates of restenosis and thus represent the current gold standard in percutaneous coronary interventions. While DESs have been around for over a decade, the drug release mechanism(s) and the uptake into the arterial wall are still poorly understood.

The problem of modelling drug release from arterial stents was presented at the fourth UK Mathematics in Medicine Study Group (see Green *et al.*, 2005) and many publications on this topic have appeared in the literature to date. One of the most important aspects in the performance of DESs is the drug release profile. Owing to the expense and time associated with *in vivo* experiments, stent manufacturers routinely test the release of drug from their stents in an *in vitro* environment. While this is unlikely to replicate the *in vivo* situation, where flowing blood, pulsatility, wound healing, proliferation, migration of cells and complex uptake/binding no doubt all play some part, it nonetheless provides the manufacturer with an idea of the shape of the release profile and allows for comparison between different stent designs. Furthermore, it allows the manufacturer to test the quality of the product and the repeatability of the release profile. While stent manufacturers primarily use empirically based methods, when coupled with appropriate modelling, the *in vitro* experiments may provide insights into the mechanism(s) of release: this is a topic of some controversy in the literature with diffusion, dissolution, polymer swelling and degradation all of which are cited as possible release mechanisms. Several authors have attempted to model the drug release from these devices. For example, referring to polymer-coated DESs which is the focus of this article, Zhao *et al.* (2012) presented an analytic solution of a cylindrical diffusion model to describe the experimental drug release of everolimus from a Dynalink-E stent while Hossainy and Prabhu (2008) presented a mathematical model for predicting the drug release from a DES coating, based on two discrete modes of transport. Most models assume that Fickian diffusion plays an important role in the release process. Siepmann and Siepmann (2012) in their review on modelling of diffusion-controlled drug delivery provide a series of analytical solutions for drug release from reservoir and monolithic drug delivery systems, some of which may be applied to DESs under certain assumptions. Many of their solutions are early or late time approximations, and in some cases steady-state solutions. A number of authors have focussed on drug release from biodegradable polymers: we do not consider such polymers in this article, but the reader is referred to Formaggia *et al.* (2010), Rossi *et al.* (2012) and Fredenberg *et al.* (2011).

In reality, of course, the stent and the arterial wall are a coupled system and this has also received much attention in the literature, with most of the models necessarily requiring to be solved numerically due to the complexity of the problem. One exception is the model of Pontrelli and de Monte (2010) who consider diffusion-based drug release from the stent coupled with a convection–diffusion equation in the arterial wall which also accounts for drug consumption via a linear reaction. They obtain an analytical solution through separation of variables and use this to show the effect of the transmural velocity, drug metabolism and the amount of drug in the tissue. More recently, Pontrelli *et al.* (2013a) presented a semi-analytical expression for the drug concentration and mass in each layer of the arterial wall for the case where the drug must dissolve in the polymer before it can diffuse. Models which incorporate more sophisticated binding/reaction terms includes those of McGinty *et al.* (2010), McGinty *et al.* (2013), Horner *et al.* (2010) and Abraham *et al.* (2013), who all assume an equilibrium reaction. While McGinty *et al.* (2013) made some analytical progress, the other authors employed numerical techniques to solve their equations. Perhaps the most sophisticated model is that of Tzafriri *et al.* (2012), who provide a second-order saturable reversible binding model: this model not only requires to be solved numerically, but also includes a number of parameters which are difficult to measure experimentally.

In most of the models described above, many parameters characterizing the system are required to be measured, thus posing a great challenge to experimentalists. These include drug diffusion coefficients and dissolution constants in the stent polymer coating as well as drug diffusion coefficients, binding/uptake rates and the magnitude of the transmural convection in the arterial wall. Values for these parameters are typically taken from the available data in the literature which encompasses a great number of studies using different experimental species (for example, rabbit or pig). Furthermore the experimental techniques are often quite different. For example, many experiments are conducted *in vitro* when the transmural velocity has necessarily been neglected and in many cases 'lumped diffusion coefficients', which inherently include such effects as convection and binding, are measured. Thus great care must be taken to ensure the accurate estimation of the model parameters. In this paper we attempt to address this problem by providing analytical solutions which, when compared with appropriate experiments, may allow the various parameters of the system to be estimated via the inverse problem. Of course, the inverse approach we propose here to estimate model parameters may also be applied to other drug delivery systems, for example, delivery by drug-filled balloons and transdermal patches (see, for example, Stark *et al.*, 2013; Pontrelli *et al.*, 2013b).

This paper is organized as follows. Firstly we present a collection of models to describe diffusion- and dissolution-based release of drug from polymer-coated arterial stents in an *in vitro* environment. The boundary conditions have been chosen to reflect the conditions maintained in the experiments. All of the models are solved analytically, providing user-friendly solutions which can be quickly compared with observed release profiles. It is intended that when used in conjunction with appropriate experimental data, these solutions should help us to clarify the release mechanism(s) and thus aid in the development of DESs. By consideration of the inverse problem, the various parameters characterizing the release may be determined. We then proceed to consider a model of drug transport through the arterial wall. This model too is solved analytically, providing three interesting characterizations of the solution. We demonstrate the equivalence of these solutions and, by comparison with experimental data via an inverse problem, attempt to uncouple and estimate both the tissue drug diffusion coefficient and convection parameter.

## 2. Diffusion-based models of drug release

Most of the modelling of drug release from arterial stents in the literature has thus far been concerned with first generation DESs. These stents typically comprise a stainless steel platform with a drug containing polymer coating attached to the stent struts (Tzafriri *et al.*, 2012; Stefanini & Holmes, 2013). The philosophy behind this design was not only to allow the drug to be released gradually so as to avoid toxic levels of drug initially, but also to permit sustained delivery over many weeks. The drugs used (sirolimus and paclitaxel) are lipophilic and are able to inhibit SMC proliferation and migration. Drug release from these stents has been modelled as a diffusion-dominated process (see, for example, McGinty *et al.*, 2010; Pontrelli and de Monte, 2010), with the drug concentration in the polymer $C_p$ satisfying a diffusion equation with drug diffusion coefficient $D_p$. Several simplifying assumptions are made. Firstly, it is assumed that the device geometry is that of a thin film of thickness $L_p$ with no edge effects so that the modelling may be restricted to one dimension. The diffusion of the drug in the polymer is thus considered to be isotropic and it may be assumed that the diffusion coefficient is independent of time, space and concentration. Furthermore, the initial drug concentration is usually taken to be uniform. For the case of *in vitro* drug release a zero flux boundary condition is normally assumed at the impermeable stent boundary and either an infinite sink or Robin-type boundary condition at the interface with the release medium. If the release medium volume is sufficiently large and is replenished between sampling times then the infinite sink condition is most appropriate, whereas if the concentration of drug in the release medium is allowed to build up, the Robin-type condition is preferred. The model is as follows:

$$\frac{\partial C_p}{\partial t}(x,t) = D_p \frac{\partial^2 C_p}{\partial x^2}(x,t), \quad 0 < x < L_p, \quad t > 0, \tag{2.1}$$

subject to

$$C_{\rm p}(x,0)=C^0, \quad 0\leqslant x\leqslant L_{\rm p}, \tag{2.2}$$

$$-D_{\rm p}\frac{\partial C_{\rm p}}{\partial x}(0,t)=0, \quad t>0, \tag{2.3}$$

$$C_{\rm p}(L_{\rm p},t)=0 \quad \text{or} \quad -D_{\rm p}\frac{\partial C_{\rm p}}{\partial x}(L_{\rm p},t)=\gamma C_{\rm p}(L_{\rm p},t), \quad t>0, \tag{2.4 a,b}$$

where $\gamma$ is some constant of proportionality with dimensions $\text{ms}^{-1}$. Of course (2.4a) can be obtained from (2.4b) by letting $\gamma\to\infty$ which physically means that the flux across the interface is extremely rapid. For the case of the infinite sink boundary condition, the system (2.1–2.4a) are readily solved using the method of Laplace transforms (or otherwise) to provide (see, e.g. Crank, 1975)

$$C_{\rm p}(x,t)=-\frac{4C^0}{\pi}\sum_{n=1}^{\infty}\frac{(-1)^n}{2n-1}\cos\left(\frac{(2n-1)\pi x}{2L_{\rm p}}\right)\exp\left\{\frac{-(2n-1)^2\pi^2D_{\rm p}t}{4L_{\rm p}^2}\right\}. \tag{2.5}$$

We can obtain the mass of drug per unit area in the polymer, $M(t)$, by integrating $C_{\rm p}(x,t)$ over $L_{\rm p}$. From this it is straightforward to show that the cumulative fraction of drug released, $M_{\rm frac}(t)=1-M(t)/M_0$, is

$$M_{\rm frac}(t)=1-\frac{8}{\pi^2}\sum_{n=1}^{\infty}\frac{1}{(2n-1)^2}\exp\left\{\frac{-(2n-1)^2\pi^2D_{\rm p}t}{4L_{\rm p}^2}\right\}, \tag{2.6}$$

where $M_0=L_{\rm p}C^0$ has been assumed. For the case where the concentration is allowed to rise in the release medium (i.e. employing (2.4b) rather than (2.4a)), we can again utilize Laplace transforms along with the Residue Theorem to provide the solution

$$C_{\rm p}(x,t)=2\gamma C^0\sum_{n=1}^{\infty}\frac{\exp\{-D_{\rm p}\alpha_n^2t\}\cos(\alpha_nx)}{\alpha_n[(D_{\rm p}+\gamma L_{\rm p})\sin(\alpha_nL_{\rm p})+\alpha_nD_{\rm p}L_{\rm p}\cos(\alpha_nL_{\rm p})]}, \tag{2.7}$$

where $\alpha_n$ are the roots of

$$-D_{\rm p}\alpha\sin(\alpha L_{\rm p})+\gamma\cos(\alpha L_{\rm p})=0.$$

In this case the cumulative fraction of drug released turns out to be

$$M_{\rm frac}(t)=1-\frac{2\gamma}{L_{\rm p}}\sum_{n=1}^{\infty}\frac{\exp\{-D_{\rm p}\alpha_n^2t\}\sin(\alpha_nL_{\rm p})}{\alpha_n^2[(D_{\rm p}+\gamma L_{\rm p})\sin(\alpha_nL_{\rm p})+\alpha_nD_{\rm p}L_{\rm p}\cos(\alpha_nL_{\rm p})]}. \tag{2.8}$$

In Fig. 1 we compare the release profiles obtained from Equations (2.6) and (2.8). We assume the typical values of diffusion coefficient and polymer thickness of $D_{\rm p}=10^{-16}\,\text{m}^2\,\text{s}^{-1}$ and $L_{\rm p}=10^{-5}\,\text{m}$, respectively (McGinty *et al.*, 2010). The solution (2.8) is plotted for three values of $\gamma$, $10^{-10}\,\text{ms}^{-1}$, $10^{-11}\,\text{ms}^{-1}$ and $10^{-12}\,\text{ms}^{-1}$. It is evident that for the case where the drug is allowed to rise in the release medium, the release of the drug can be considerably slowed down.

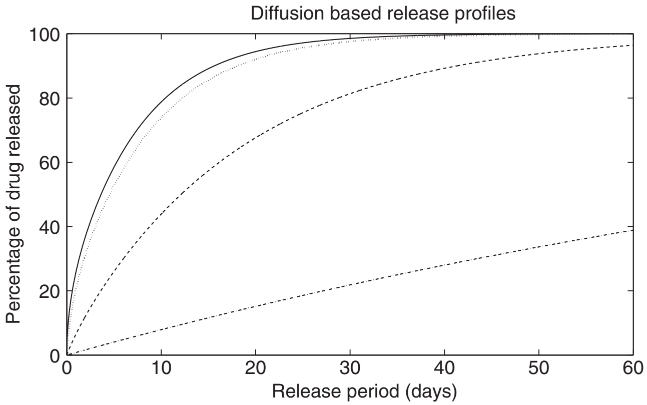

FIG. 1. Comparison of release profiles for the cases of release medium being replenished (infinite sink) and drug rising in the release medium (Robin boundary condition). The top profile displays the solution (2.6), while as we move down, the profiles display the solution of (2.8) with $\gamma = 10^{-10}\,\mathrm{ms}^{-1}$, $\gamma = 10^{-11}\,\mathrm{ms}^{-1}$ and $\gamma = 10^{-12}\,\mathrm{ms}^{-1}$.

## 3. Diffusion–dissolution-based models of drug release

In the preceding section, we considered models of drug release whereby the drug was assumed to be free to diffuse. However, this need not be the case. In fact it is common for the drug to be loaded on the polymer in such a way that the initial concentration exists in two forms: crystalline (bound) and free (unbound). In this case the drug needs to dissolve before it can freely diffuse. This can be achieved by the ingress of fluid into the polymer after placement in the release medium. In this section we describe three models which incorporate aspects of dissolution as well as diffusion. In all of these models we assume an infinite sink boundary condition.

### 3.1 *Instantaneous dissolution model*

In this case we assume that the initial concentration of drug in the polymer exceeds the solubility limit, say $C_s$ (assumed to be constant). Thus drug exists in a crystalline form and is permitted only to diffuse after it has dissolved. Where the initial drug concentration is below the solubility limit, $C^0 < C_s$, the dissolved drug is free to diffuse. In this case the solutions given by (2.5–2.6) describe the concentration profile and cumulative fraction of drug released. However, if $C^0 > C_s$ then there is an excess of drug which is immobile and must dissolve before diffusion can commence. This problem was first considered by Higuchi (1961) who derived an expression for the amount of drug absorbed at time $t$ per unit area. He obtained his solution from geometrical considerations and Fick's law. A more mathematical approach has been considered by, among others, Paul & McSpadden (1976). However, these approaches derive a solution that is only valid until the time at which the concentration of drug throughout the polymer falls below the solubility, say $t = t^{\star}$. Here we provide the complete solution which is valid for all time.

Suppose that there is a small region immediately adjacent to the release medium boundary where the concentration of drug is less than the solubility, i.e. it is permitted to diffuse. Suppose further that to the left of the region, the drug concentration is above solubility. Let there be a moving boundary between these two regions whose time varying location is given by $x = s(t)$. As drug is released from the polymer the moving boundary tracks back from the (initially fixed) boundary with the release medium to the impermeable stent boundary. We assume that the dissolution is instantaneous so that the concentration on the moving boundary is equal to the solubility. Then the model is

$$C_{\mathrm{p}}(x,t) = C^0, \quad 0 < x < s(t), \;\; 0 < t < t^\star, \tag{3.1}$$

$$\frac{\partial C_{\mathrm{p}}}{\partial t}(x,t) = D_{\mathrm{p}} \frac{\partial^2 C_{\mathrm{p}}}{\partial x^2}(x,t) \quad s(t) < x < L_{\mathrm{p}}, \;\; 0 < t < t^\star, \tag{3.2}$$

$$C_{\mathrm{p}}(s(t),t) = C_s, \quad 0 < t < t^\star, \tag{3.3}$$

$$C_{\mathrm{p}}(L_{\mathrm{p}},t) = 0, \quad 0 < t < t^\star, \tag{3.4}$$

$$s(0) = L_{\mathrm{p}}, \quad 0 < t < t^\star, \tag{3.5}$$

coupled with the Stefan-type boundary condition

$$-D_{\mathrm{p}} \frac{\partial C_{\mathrm{p}}}{\partial x}(s(t),t) = \frac{\mathrm{d}s}{\mathrm{d}t}(C_s - C^0). \tag{3.6}$$

The problem is readily seen to have a similarity structure. If we let $C_{\mathrm{p}}(x,t) = F(\xi)$, $s(t) = L_{\mathrm{p}} - \theta\sqrt{t}$ and $\xi = (L_{\mathrm{p}} - x)/\sqrt{t}$ then the problem (3.1–3.6) becomes

$$D_{\mathrm{p}} \frac{\mathrm{d}^2 F}{\mathrm{d}\xi^2}(\xi) = -\frac{\xi}{2}\frac{\mathrm{d}F}{\mathrm{d}\xi}(\xi), \quad 0 < \xi < \theta. \tag{3.7}$$

$$F(0) = 0, \tag{3.8}$$

$$F(\theta) = C_s, \tag{3.9}$$

$$D_{\mathrm{p}} \frac{\mathrm{d}F}{\mathrm{d}\xi}(\theta) = \frac{\theta}{2}(C^0 - C_s). \tag{3.10}$$

The solution for $t < t^\star$ is (see Vo, 2012 or Paul & McSpadden, 1976):

$$C_{\mathrm{p}}(x,t) = C_s \frac{\mathrm{erf}\,((L_{\mathrm{p}} - x)/2\sqrt{D_{\mathrm{p}} t})}{\mathrm{erf}\,(\theta/2\sqrt{D_{\mathrm{p}}})}, \quad s(t) < x < L_{\mathrm{p}}, \tag{3.11}$$

where $\theta$ is found by solving

$$2\sqrt{\frac{D_{\mathrm{p}}}{\pi}} \frac{C_s}{C^0 - C_s} = \theta \,\mathrm{erf}\left(\frac{\theta}{2\sqrt{D_{\mathrm{p}}}}\right) \exp\left\{\frac{\theta^2}{4D_{\mathrm{p}}}\right\}. \tag{3.12}$$

Note $t^\star$ is known and is given by $s(t^\star) = 0$, i.e. $t^\star = (L_{\mathrm{p}}/\theta)^2$. The expression for the cumulative fraction of drug released is found to be

$$M_{\mathrm{frac}}(t) = \frac{\sqrt{t}}{L_{\mathrm{p}} C^0} \left\{ \theta(C^0 - C_s) + \frac{2\sqrt{D_{\mathrm{p}}} C_s (1 - \exp\{-\theta^2/4D_{\mathrm{p}}\})}{\sqrt{\pi}\,\mathrm{erf}\,(\theta/2\sqrt{D_{\mathrm{p}}})} \right\}, \quad t < t^\star. \tag{3.13}$$

Now, when all of the drug in the polymer is below solubility, the form of solution changes. At $t = t^\star$, the concentration of drug in the polymer is clearly given by

$$C_{\mathrm{p}}(x,t^\star) = C_s \frac{\mathrm{erf}\,((L_{\mathrm{p}} - x)/2\sqrt{D_{\mathrm{p}} t^\star})}{\mathrm{erf}\,(\theta/2\sqrt{D_{\mathrm{p}}})}. \tag{3.14}$$

The solution for the duration of drug release is then obtained by solving (2.1), (2.3) and (2.4a) with (3.14) replacing (2.2). Proceeding to solve by separation of variables, the concentration of the drug in the polymer can be shown to be (see Appendix A)

$$C_{\mathrm{p}}(x,t)=\frac{4C_s}{\pi\,\mathrm{erf}\,(\theta/2\sqrt{D_{\mathrm{p}}})}\sum_{n=1}^{\infty}\frac{(-1)^{n+1}}{2n-1}\cos\left(\frac{(2n-1)\pi x}{2Lp}\right)\exp\left\{\frac{-Dp(2n-1)^2\pi^2}{4Lp}t\right\}$$
$$\times\,\Re\left\{\mathrm{erf}\left(\frac{(2n-1)\mathrm{i}\pi D_{\mathrm{p}}t^\star+Lp^2}{2L_{\mathrm{p}}\sqrt{D_{\mathrm{p}}t^\star}}\right)\right\}, \tag{3.15}$$

where $\Re\{z\}$ denotes the real part of $z$. The corresponding expression for the cumulative fraction of drug released for $t > t^\star$ (see Appendix A) is

$$M_{\mathrm{frac}}(t)=1-\frac{8C_s}{C^0\pi^2\,\mathrm{erf}(\theta/2\sqrt{D_{\mathrm{p}}})}$$
$$\times\sum_{n=1}^{\infty}\frac{\exp\{(-D_{\mathrm{p}}(2n-1)^2\pi^2/4L_{\mathrm{p}})t\}\Re\{\mathrm{erf}(((2n-1)\mathrm{i}\pi D_{\mathrm{p}}t^\star+L_{\mathrm{p}}^2)/2L_{\mathrm{p}}\sqrt{D_{\mathrm{p}}t^\star})\}}{(2n-1)^2}. \tag{3.16}$$

Recall that $t^\star=(L_{\mathrm{p}}/\theta)^2$ where $\theta$ is given by (3.12).

### 3.2 *Dissolution kinetics involving a first-order reaction*

In the previous case we considered a situation where the dissolution of drug from crystalline into dissolved form is instantaneous. This may be reasonable where the volume of fluid available to dissolve the drug is large. Consider now the case where the dissolution rate is finite, say $K_1$. We will now denote by $C_{\mathrm{b}}$ the bound drug which is in a crystalline form and $C_{\mathrm{f}}$ the free (dissolved) drug which is initially zero. The model in this case is

$$\frac{\partial C_{\mathrm{f}}}{\partial t}(x,t)=D_{\mathrm{p}}\frac{\partial^2 C_{\mathrm{f}}}{\partial x^2}(x,t)+K_1C_{\mathrm{b}}(t),\quad 0<x<L_{\mathrm{p}},\quad t>0, \tag{3.17}$$

$$\frac{\mathrm{d}C_{\mathrm{b}}}{\mathrm{d}t}(t)=-K_1C_{\mathrm{b}}(t),\quad 0<x<L_{\mathrm{p}},\quad t>0, \tag{3.18}$$

$$C_{\mathrm{b}}(0)=C_{\mathrm{b}}^0,\quad 0\leqslant x\leqslant L_{\mathrm{p}}, \tag{3.19}$$

$$C_{\mathrm{f}}(x,0)=0,\quad 0\leqslant x\leqslant L_{\mathrm{p}}, \tag{3.20}$$

$$-D_{\mathrm{p}}\frac{\partial C_{\mathrm{f}}}{\partial x}(0,t)=0,\quad t>0, \tag{3.21}$$

$$C_{\mathrm{f}}(L_{\mathrm{p}},t)=0,\quad t>0. \tag{3.22}$$

The solution of the system (3.17–3.22) is detailed in Appendix B and is given by

$$C_b(t) = C_b^0 \exp\{-K_1 t\}, \tag{3.23}$$

$$C_f(x,t) = \frac{4K_1C_b^0}{\pi}\sum_{n=1}^{\infty}\frac{(-1)^{n+1}\cos((2n-1)\pi x/2L_p)}{(2n-1)(K_1-(D_p/4L_p^2)(2n-1)^2\pi^2)} \times\left\{\exp\left\{\frac{-D_p}{4L_p^2}(2n-1)^2\pi^2 t\right\}-\exp\{-K_1t\}\right\}. \tag{3.24}$$

The solution for the cumulative mass of drug released is

$$M_{\text{frac}}(t) = 1-\exp\{-K_1t\}-\frac{8K_1}{\pi^2}\sum_{n=1}^{\infty}\frac{\exp\{(-D_p/4L_p^2)(2n-1)^2\pi^2 t\}-\exp\{-K_1t\}}{(2n-1)^2(K_1-(D_p/4L_p^2)(2n-1)^2\pi^2)}. \tag{3.25}$$

### 3.3 *Dissolution kinetics involving a first-order reversible reaction*

In this case we allow for a reversible reaction, i.e. the drug can dissolve from the crystalline form into free form and also from free form into crystalline form. We assume that the forward reaction rate is $K_1$ and the backward reaction is $K_2$. The model in this case is

$$\frac{\partial C_f}{\partial t}(x,t) = D_p\frac{\partial^2 C_f}{\partial x^2}(x,t)+K_1C_b(x,t)-K_2C_f(x,t), \quad 0<x<L_p, \quad t>0, \tag{3.26}$$

$$\frac{\partial C_b}{\partial t}(x,t) = -K_1C_b(x,t)+K_2C_f(x,t) \quad 0<x<L_p, \quad t>0 \tag{3.27}$$

coupled with (3.19–3.22). The solution as detailed in Appendix B is

$$C_f(x,t)=\frac{K_1C_b^0D_p\pi}{L_p^2}\sum_{j=1}^{2}\sum_{n=1}^{\infty}\frac{(-1)^n(2n-1)(s_{j_n}+K_1)^2\cos((2n-1)\pi x/2L_p)\,(\exp\{s_{j_n}t\}-\exp\{-(K_2+K_1)t\})}{s_{j_n}(s_{j_n}^2+2K_1s_{j_n}+K_1(K_2+K_1))(K_2+K_1+s_{j_n})}, \tag{3.28}$$

$$C_b(x,t)=C_b^0\exp\{-K_1t\}+\frac{K_2K_1C_b^0D_p\pi}{L_p^2} \times\sum_{j=1}^{2}\sum_{n=1}^{\infty}\left\{\frac{(-1)^n(2n-1)(s_{j_n}+K_1)^2\cos((2n-1)\pi x/2L_p)}{s_{j_n}(s_{j_n}^2+2K_1s_{j_n}+K_1(K_2+K_1))(K_2+K_1+s_{j_n})} \times\left(\frac{\exp\{s_{j_n}t\}-\exp\{-K_1t\}}{K_1+s_{j_n}}+\frac{\exp\{-(K_2+K_1)t\}-\exp\{-K_1t\}}{K_2}\right)\right\}, \tag{3.29}$$

where

$$2s_{1_n} = -\left(K_2 + K_1 + \frac{D_p}{4L_p^2}\pi^2(2n-1)^2\right) + \sqrt{(K_2 + K_1 + \frac{D_p}{4L_p^2}\pi^2(2n-1)^2)^2 - \frac{K_1 D_p}{L_p^2}\pi^2(2n-1)^2},$$

$$2s_{2_n} = -\left(K_2 + K_1 + \frac{D_p}{4L_p^2}\pi^2(2n-1)^2\right) - \sqrt{\left(K_2 + K_1 + \frac{D_p}{4L_p^2}\pi^2(2n-1)^2\right)^2 - \frac{K_1 D_p}{L_p^2}\pi^2(2n-1)^2}. \tag{3.30}$$

The cumulative fraction of drug released is then given by

$$\begin{aligned} M_{\text{frac}}(t) = 1 - \exp\{-K_1 t\} \\ + \frac{2K_2 K_1 D_p}{L_p^2} \sum_{j=1}^{2} \sum_{n=1}^{\infty} \frac{(s_{j_n} + K_1)^2 \left(\frac{\exp\{s_{j_n} t\} - \exp\{-K_1 t\}}{K_1 + s_{j_n}} + \frac{\exp\{-(K_2+K_1)t\} - \exp\{-K_1 t\}}{K_2}\right)}{s_{j_n}(s_{j_n}^2 + 2K_1 s_{j_n} + K_1(K_2 + K_1))(K_2 + K_1 + s_{j_n})} \\ + \frac{2K_1 D_p}{L_p^2} \sum_{j=1}^{2} \sum_{n=1}^{\infty} \frac{(s_{j_n} + K_1)^2 (\exp\{s_{j_n} t\} - \exp\{-(K_2 + K_1)t\})}{s_{j_n}(s_{j_n}^2 + 2K_1 s_{j_n} + K_1(K_2 + K_1))(K_2 + K_1 + s_{j_n})}. \end{aligned} \tag{3.31}$$

### 3.4 *Graphical results*

In this section we compare the release profiles obtained from the solutions to the diffusion–dissolution models described in Sections 3.1–3.3. For each of the plots we assume typical values of the diffusion coefficient and the polymer thickness, $D_p = 10^{-16}$ m$^2$ s$^{-1}$ and $L_p = 10^{-5}$ m, respectively, as in Section 2. Figure 2 plots the cumulative percentage of drug released (Equations (3.13) and (3.16)) for the instantaneous dissolution model described in Section 3.1. We consider four different ratios of initial drug concentration to solubility, $C^0/C_s = 10, 5, 2, 1$. Figure 2 demonstrates that by increasing the fraction $C^0/C_s$, we can significantly prolong the duration of release. We note that when $C^0/C_s = 1$, i.e. when the initial drug concentration is not above solubility, we obtain a release profile identical to that provided by (2.6) as expected.

Figure 3 plots the cumulative percentage of drug released (Equation (3.25)) for the first-order reaction model described in Section 3.2. We consider three different values of $K_1$, namely $10^{-4}$ s$^{-1}$, $10^{-5}$ s$^{-1}$ and $10^{-6}$ s$^{-1}$, which correspond to Damkohler numbers ($Da = L_p^2 K_1/D_p$) of 100, 10 and 1. We demonstrate that, as we reduce the Damkholer number (by reducing the value of $K_1$), the release profile can be significantly slowed down as diffusion becomes faster than reaction: in this case the system is thus reaction-limited. In Fig. 4, we display the cumulative percentage of drug released (Equation (3.31)) for the first-order reversible reaction model described in Section 3.3. We choose $K_1 = 10^{-6}$ s$^{-1}$ and consider four different ratios of $K_1/K_2$, namely $100, 10, 1, 0.1$ and demonstrate that in the case of a reversible reaction, the release can also be significantly slowed down. Furthermore, we note that for large $K_1/K_2$, the profile approaches that of the non-reversible reaction model. We note, too, that there is a qualitative difference in the shape of the profile of the three different models.

## 4. Mechanism of drug release: the inverse problem

In Sections 2 and 3 we provided a series of analytical solutions to models which encompass diffusion- and dissolution-based drug release. Here we test our models on *in vitro* experimental data that we have

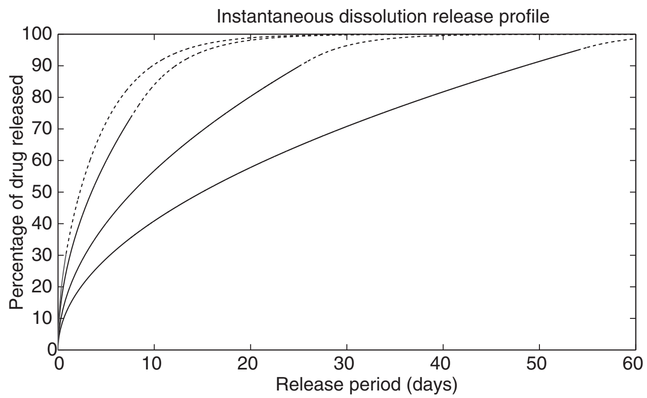

FIG. 2. Release profiles for the case of instantaneous dissolution. From top to bottom profiles $C^0/C_s$ increases from 1 to 2, 5 and then 10. Solid lines indicate $t < t^\star$ while dotted lines indicate $t > t^\star$.

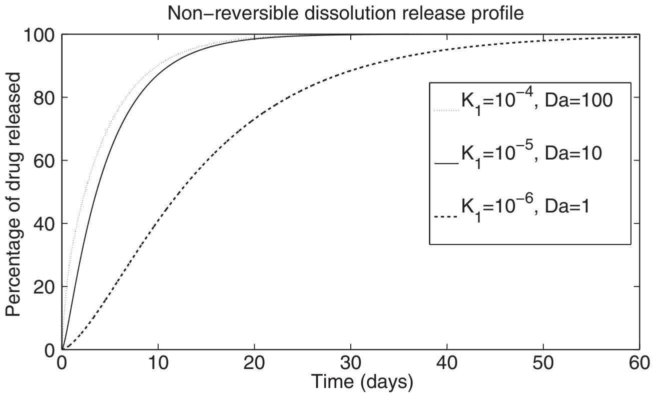

FIG. 3. Release profiles for the case of non-reversible dissolution. As we reduce the Damkohler number, *Da*, we observe that the release is slowed down.

obtained in our laboratory to try to ascertain the mechanism of release in a commercially available DES, as well as estimating the model parameter(s) via the inverse problem. The experiments involved measuring drug release from the Cypher DES. We have previously reported on the drug release from the Cypher DES (McGinty *et al.*, 2013). Briefly, the experiments consisted of placing four Cypher DESs in separate sealed glass vials containing physiological release medium (phosphate buffered saline:ethanol (90:10)). The experiments were carried out at 37°C. At several time points up to 60 days, each stent was removed and placed in a separate vial containing fresh release medium (to maintain perfect sink conditions), with the mass of drug in the original solution subsequently quantified using UV-spectroscopy. Solving an inverse problem consists of adjusting the parameters of a model function so as to best fit the data set. In this case, the data set should be the experimentally measured mass of drug released at various times. Adopting a least squares approach (and solving the resulting nonlinear equations via Newton's method), we find that drug release from the Cypher stent is best described by the diffusion only model given by equations (2.1–2.4a). The best fitting value of the diffusion coefficient is

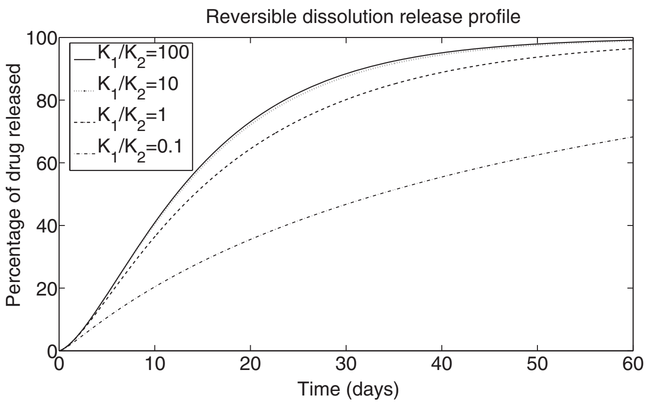

FIG. 4. Release profiles for the case of reversible dissolution. Here $K_1 = 10^{-6}\,\mathrm{s}^{-1}$ and we vary the value of $K_2$. For $K_1/K_2 \geqslant 1$, the effect of the reversible dissolution is relatively small, as expected. However, when $K_1/K_2 < 1$ so that the backward reaction is faster than the forward reaction, the release may be significantly slowed down.

$D_{\mathrm{p}} = 6.3 \times 10^{-17}\,\mathrm{m}^2\,\mathrm{s}^{-1}$. Figure 5 displays a comparison between the experimentally measured cumulative percentage of drug released, and the solution of (2.6). The good agreement between the model and the experiments serves to demonstrate that diffusion is the dominant mechanism of release, at least for this particular stent. For the case of the instantaneous diffusion model, the best fit was obtained when $C^0/C_s$=1, i.e. when the initial drug concentration is not above solubility. In this case the solution is equivalent to (2.6). For the first-order reaction model, the best fit was obtained when the reaction rate, $K_1$, was so large that the dissolution could be considered instantaneous. Similarly, for the first-order reversible reaction model, the best fit was obtained when $K_1$ was very large and $K_2$ very small, again suggesting dissolution can be regarded as instantaneous, while also indicating a negligible backward reaction.

## 5. Estimating physiological transport parameters

In many physiological processes, it is extremely difficult in practice to measure diffusion coefficients. This is in part, at least, because there is often a small but significant convection flow; this is particularly true near to arterial walls where there is a transmural pressure gradient and a consequent transmural flow. In addition, the species diffusing may be consumed or lost from the system through such vehicles as the *vasa vasorum*. In this section we indicate how a simple mathematical approach may allow the diffusion coefficient to be uncoupled from these other effects.

### 5.1 *Drug transport through the arterial wall*

Consider the initial value problem

$$\frac{\partial C_{\mathrm{T}}}{\partial t}(x,t) + v\frac{\partial C_{\mathrm{T}}}{\partial x}(x,t) = D_{\mathrm{T}}\frac{\partial^2 C_{\mathrm{T}}}{\partial x^2}(x,t) - \alpha C_{\mathrm{T}}(x,t) \tag{5.1}$$

subject to

$$C_{\mathrm{T}}(x,0) = 0, \quad C_{\mathrm{T}}(0,t) = C^0, \quad t \geqslant 0, \ C_{\mathrm{T}}(x,t) \text{ bounded for all } x, t,$$

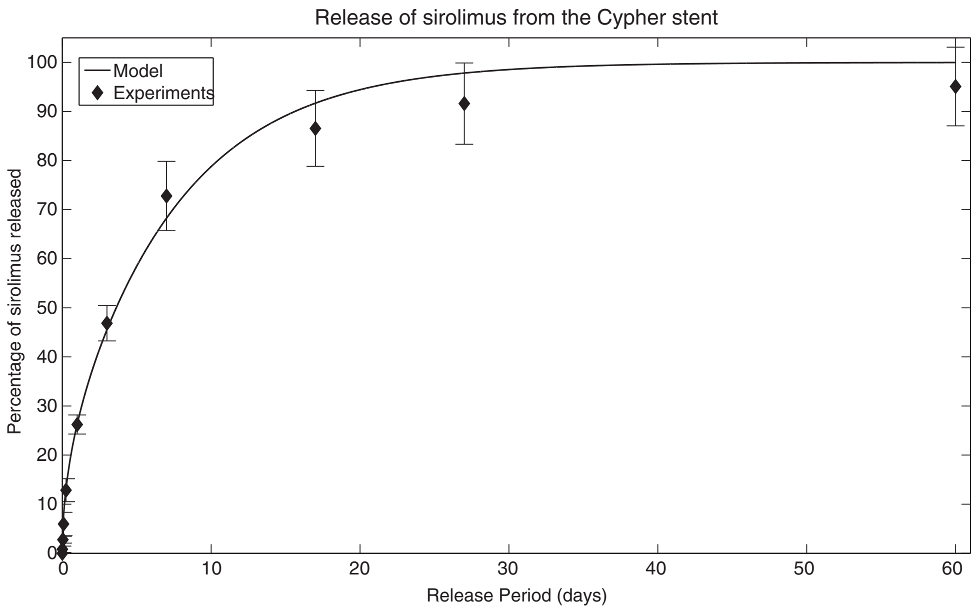

FIG. 5. Comparison between *in-vitro* experimental data and diffusion based model of drug release from the Cypher stent (McGinty *et al.*, 2013).

where $C_T$ is the drug concentration in the tissue, $C^0$ some constantly applied concentration, $v$ the magnitude of the transmural velocity, $D_T$ the diffusion coefficient in the tissue and drug is removed from the system in proportion to $\alpha$. For the case of the drug-eluting stent, the tissue is considered to be the region $(0 < x < \infty)$ and while a constant applied concentration of drug at the polymer/media interface is somewhat unrealistic, it may, nonetheless provide a good approximation for early times. A more comprehensive model has been considered by Pontrelli and de Monte (2009), where the equation for drug transport in the tissue (5.1) has been coupled with a diffusion equation for the release of drug from the coating. Here, to effect comparisons with *ex vivo* experimental data, we restrict our attention to the equation in the tissue.

Taking Laplace transforms of (5.1) and making use of the boundary conditions leads to

$$\bar{C_T}(x,s) = C^0 \exp\left\{\frac{v}{2D_T}x\right\}\frac{1}{s}\exp\left\{-\frac{x}{\sqrt{D_T}}\sqrt{s+\frac{v^2}{4D_T}+\alpha}\right\}. \tag{5.2}$$

Now let $x/\sqrt{D_T} = \sqrt{a}$ and $(v^2/4D_T) + \alpha = b$, so that (5.2) can be written in the more concise form

$$\bar{C_T}(x,s) = C^0 \exp\left\{\frac{v}{2D_T}x\right\}\frac{1}{s}\exp\{-\sqrt{a(s+b)}\}. \tag{5.3}$$

Applying Lemmas 1 and 2 (as detailed in Appendix C) directly provides two forms of the solution:

$$C_T(x,t) = \frac{C^0}{2}\exp\left\{\frac{v}{2D_T}x\right\} \times \left[\begin{array}{l} \exp\left\{-x\sqrt{\dfrac{v^2}{4D_T^2}+\dfrac{\alpha}{D_T}}\right\}\operatorname{erfc}\left(-\sqrt{\left(\dfrac{v^2}{4D_T}+\alpha\right)t}+\dfrac{x}{2\sqrt{D_T t}}\right) \\ +\exp\left\{x\sqrt{\dfrac{v^2}{4D_T^2}+\frac{\alpha}{D_T}}\right\}\operatorname{erfc}\left(\sqrt{\left(\dfrac{v^2}{4D_T}+\alpha\right)t}+\dfrac{x}{2\sqrt{D_T t}}\right)\end{array}\right], \tag{5.4}$$

$$C_T(x,t) = C^0\exp\left\{\frac{v}{2D_T}x\right\} \times \left[\begin{array}{l} \exp\left\{-\left(\dfrac{v^2}{4D_T}+\alpha\right)t\right\}\operatorname{erfc}\left(\dfrac{1}{2}\dfrac{x}{\sqrt{D_T t}}\right) \\ +\left(\dfrac{v^2}{4D_T}+\alpha\right)\displaystyle\int_0^t \exp\left\{-\left(\dfrac{v^2}{4D_T}+\alpha\right)\tau\right\}\operatorname{erfc}\left(\dfrac{1}{2}\dfrac{x}{\sqrt{D_T \tau}}\right)\mathrm{d}\tau\end{array}\right], \tag{5.5}$$

where erfc is the complementary error function. Clearly, letting $v,\alpha \to 0$ in (5.4) and (5.5), returns the well known solution for the diffusion equation, that is

$$C_T(x,t) = C^0\operatorname{erfc}\left(\frac{x}{2\sqrt{D_T t}}\right). \tag{5.6}$$

The two forms of solution, (5.4) and (5.5), can be shown to be equivalent (see Appendix D) and further, a third equivalent form of solution is given by

$$C_T(x,t) = \frac{2C^0}{\sqrt{\pi}}\exp\left\{\frac{v}{2D_T}x\right\}\int_{x/2\sqrt{D_T t}}^{\infty}\exp\left\{-\frac{x^2}{4u^2}\left(\frac{v^2}{4D_T^2}+\frac{\alpha}{D_T}\right)-u^2\right\}\mathrm{d}u. \tag{5.7}$$

### 5.2 *Inverse problem*

The motivation for developing a solution to (5.1) was to allow the various model parameters to be inferred via the inverse problem. The data set, in this case, should be a series of experimental tissue concentration profiles where the drug transport is governed by convection as well as diffusion and drug is lost from the system.

The data we would require to fit to our analytic solution do not appear to be available at present in the literature. However, Creel *et al.* (2000) provide concentration profiles based on experiments which, while admittedly do not account for drug loss (and thus render our parameter $\alpha$ redundant), do still allow us to estimate $D_T$ and $v$. Figure 6 shows experimentally measured tissue paclitaxel concentration, normalized with respect to the applied endovascular concentration. The data are obtained from experiments where arterial samples were perfused *ex vivo* for 15 min, 1 h and 4 h with a physiological transmural pressure gradient. Paclitaxel was applied to the endovascular aspect of the artery in buffer solution and drug distribution determined through en-face cryosectioning. In applying a least squares approach, starting guesses of $v = 10^{-8}\,\mathrm{ms}^{-1}$ and $D_T = 10^{-12}\,\mathrm{m}^2\,\mathrm{s}^{-1}$ (McGinty *et al.*, 2010) result in convergence of the least squares method to the values $v = 1.8\times10^{-7}\,\mathrm{ms}^{-1}$ and $D_T = 2.1\times10^{-13}\mathrm{m}^2\,\mathrm{s}^{-1}$. This result is particularly interesting, since despite an initial starting guess an order of magnitude lower,

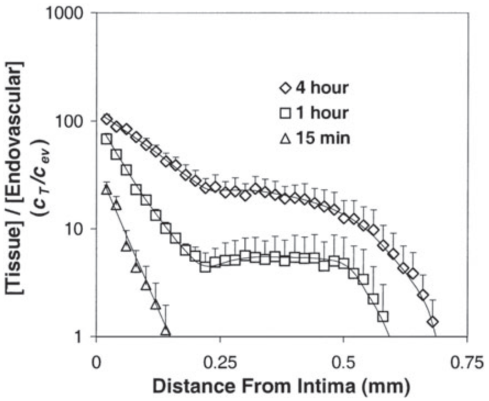

FIG. 6. Tissue concentration normalized by the endovascularly applied concentration as a function of time, in the presence of physiological transmural hydrostatic pressure gradient. Reproduced from Creel, C. J., Lovich, M. A. & Edelman, E. R. (2000) Arterial paclitaxel distribution and deposition. Circ. Res., 86, 874–884 with permission from Lippincott Williams & Wilkins. Promotional and commerical use of the material in print, digital or mobile device format is prohibited without the permission from the publisher Lippincott Williams & Wilkins. Please contact journalpermissions@lww.com for further information.

the parameter $v$ converges to a value an order of magnitude higher while the parameter $D_{\mathrm{T}}$ converges to a value an order of magnitude lower. The initial guesses along with an assumed arterial thickness of the order $10^{-4}$ m (McGinty *et al.*, 2010), results in a Peclet number $\mathcal{O}(1)$. However, the resulting Peclet number from the least squares analysis is $\mathcal{O}(100)$. This only serves to demonstrate the importance of accurate parameter estimation. While we have only been able to estimate $v$ and $D_{\mathrm{T}}$ from the experimental data available, the parameter $\alpha$ could also in principle be inferred if more appropriate experimental data were available.

## 6. Conclusions

This paper has been concerned with release mechanisms and parameter estimation in drug-eluting stent systems. Analytical solutions were obtained for mathematical models that were diffusion- and diffusion-dissolution based. In addition, analytical solutions were also provided for dissolution kinetics involving first-order reversible and non-reversible reactions. Experiments were performed *in vitro* and the subsequent data employed, via an inverse problem, to determine which of the models best fitted the *in vitro* data. It was found that the simplest model, involving only diffusion, provided the best description of *in vitro* drug release from the Cypher stent. However, it is anticipated that the other models developed in this paper may well provide a better description of the release from different stent platforms. Finally, a simple model of the transport of drug through the arterial wall was formulated; its analytical solution was then employed using the existing data from Creel *et al.* (2000) to determine the underlying diffusion coefficient and the transmural velocity through the arterial wall.

## Acknowledgement

We would like to thank Doctor Simon Kennedy (The Institute of Cardiovascular and Medical Sciences, University of Glasgow) and Professor Keith Oldroyd (Consultant Interventional Cardiologist, Golden Jubilee National Hospital, Glasgow) for their helpful suggestions and advice.

## Funding

This research was supported by the funding provided by EPSRC under grant number EP/J007242/1. The work of S.M. is also funded by a Carnegie Scholarship. Funding to pay the Open Access publication charges for this article was provided by the University of Strathclyde using funds allocated under the RCUK Open Access block grant (2013/2014).

## Appendix A

In this appendix we consider the model defined in Section 3.1 and derive the expressions for the concentration of drug in the polymer and the cumulative fraction of drug released when the concentration of drug in the polymer has dropped below the solubility. The model is stated as

$$\frac{\partial C_{\mathrm{p}}}{\partial t}(x,t) = D_{\mathrm{p}}\frac{\partial^2 C_{\mathrm{p}}}{\partial x^2}(x,t), \quad 0<x<L_{\mathrm{p}}, \quad t>t^{\star}, \tag{A.1}$$

$$-D_{\mathrm{p}}\frac{\partial C_{\mathrm{p}}}{\partial x}(0,t) = 0, \quad t>t^{\star}, \tag{A.2}$$

$$C_{\mathrm{p}}(L_{\mathrm{p}},t) = 0, \quad t>t^{\star}, \tag{A.3}$$

$$C_{\mathrm{p}}(x,t^{\star}) = C_s\frac{\mathrm{erf}\,((L_{\mathrm{p}}-x)/2\sqrt{D_{\mathrm{p}}t^{\star}})}{\mathrm{erf}\,(\theta/2\sqrt{D_{\mathrm{p}}})}, \quad 0<x<L_{\mathrm{p}}. \tag{A.4}$$

Separation of variables results in the solution

$$C_{\mathrm{p}}(x,t) = \sum_{n=1}^{\infty} B_n \cos\left(\frac{(n-1/2)\pi x}{L_{\mathrm{p}}}\right)\exp\left\{-D_{\mathrm{p}}\left(\frac{(n-1/2)\pi}{L_{\mathrm{p}}}\right)^2 t\right\}, \tag{A.5}$$

where $B_n$ $(n=1,2,\ldots)$ are constants to be determined. Imposing (A.4), multiplying through by $\cos\,((m-1/2)\pi x/L_{\mathrm{p}})$ and integrating from 0 to $L_{\mathrm{p}}$ provides:

$$\frac{C_s}{\mathrm{erf}\left(\frac{\theta}{2\sqrt{D_{\mathrm{p}}}}\right)}\int_0^{L_{\mathrm{p}}} \mathrm{erf}\left(\frac{L_{\mathrm{p}}-x}{2\sqrt{D_{\mathrm{p}}t^{\star}}}\right)\cos\left(\frac{(m-1/2)\pi x}{L_{\mathrm{p}}}\right)\mathrm{d}x$$
$$= \sum_{n=1}^{\infty} B_n \exp\left\{-D_{\mathrm{p}}\left(\frac{(n-1/2)\pi}{L_{\mathrm{p}}}\right)^2 t^{\star}\right\}\int_0^{L_{\mathrm{p}}}\cos\left(\frac{(n-1/2)\pi x}{L_{\mathrm{p}}}\right)\cos\left(\frac{(m-1/2)\pi x}{L_{\mathrm{p}}}\right)\mathrm{d}x. \tag{A.6}$$

Consider firstly the integral on the left-hand side of (A.6):

$$\int_0^{L_p} \mathrm{erf}\left(\frac{L_p - x}{2\sqrt{D_p t^\star}}\right) \cos\left(\frac{(m-1/2)\pi x}{L_p}\right) \mathrm{d}x$$

$$= \frac{2}{\sqrt{\pi}} \int_0^{L_p} \int_0^{(L_p - x)/2\sqrt{D_p t^\star}} \exp\{-z^2\}\, \mathrm{d}z \cos\left(\frac{(m-1/2)\pi x}{L_p}\right) \mathrm{d}x$$

$$= \frac{2L_p}{(m-1/2)\pi^{3/2}} \int_0^{L_p/2\sqrt{D_p t^\star}} \exp\{-z^2\} \sin\left((m-1/2)\pi - \frac{2(m-1/2)\pi}{L_p}\sqrt{D_p t^\star} z\right) \mathrm{d}z, \qquad \text{(A.7)}$$

on interchanging the integrals and performing the internal integration. Writing sine in terms of cosine in (A.7) and performing the integration results in

$$\frac{2L_p}{(m-1/2)\pi^{3/2}} \int_0^{L_p/2\sqrt{D_p t^\star}} \exp\{-z^2\} \sin\left((m-1/2)\pi - \frac{2(m-1/2)\pi}{L_p}\sqrt{D_p t^\star} z\right) \mathrm{d}z$$

$$= \frac{2L_p(-1)^{m+1}}{(m-1/2)\pi^{3/2}} \int_0^{L_p/2\sqrt{D_p t^\star}} \exp\{-z^2\} \cos\left(\frac{(2m-1)\pi}{L_p}\sqrt{D_p t^\star} z\right) \mathrm{d}z$$

$$= \frac{(-1)^{m+2} L_p}{(2m-1)\pi} \exp\left\{-\frac{(m-1/2)^2 \pi^2 D_p t^\star}{L_p^2}\right\} \left\{ \begin{array}{l} \mathrm{erf}\left(\frac{(2m-1)\mathrm{i}\pi D_p t^\star - L_p^2}{2L_p\sqrt{D_p t^\star}}\right) \\ -\,\mathrm{erf}\left(\frac{(2m-1)\mathrm{i}\pi D_p t^\star + L_p^2}{2L_p\sqrt{D_p t^\star}}\right) \end{array} \right\}. \qquad \text{(A.8)}$$

Thus, returning to (A.6), and making use of orthogonality:

$$B_n = \frac{2C_s(-1)^{n+2}}{(2n-1)\pi\ \mathrm{erf}\,(\theta/2\sqrt{D_p})} \left\{ \mathrm{erf}\left(\frac{(2n-1)\mathrm{i}\pi D_p t^\star - L_p^2}{2L_p\sqrt{D_p t^\star}}\right) - \mathrm{erf}\left(\frac{(2n-1)\mathrm{i}\pi D_p t^\star + L_p^2}{2L_p\sqrt{D_p t^\star}}\right) \right\}. \qquad \text{(A.9)}$$

Noting that

$$\mathrm{erf}\,(-\bar{z}) - \mathrm{erf}\,(z) = -2\Re\{\mathrm{erf}\,(z)\},$$

we may write (A.9) as

$$B_n = \frac{4C_s(-1)^{n+1}}{(2n-1)\pi\ \mathrm{erf}\,(\theta/2\sqrt{D_p})} \Re\left\{ \mathrm{erf}\left(\frac{(2n-1)\mathrm{i}\pi D_p t^\star + L_p^2}{2L_p\sqrt{D_p t^\star}}\right) \right\}. \qquad \text{(A.10)}$$

Substitution of (A.10) into (A.5) provides the solution as (3.15). Now, the cumulative fraction of drug released is given by

$$M_{\mathrm{frac}}(t) = 1 - \frac{M(t)}{M_0}, \qquad \text{(A.11)}$$

where $M(t) = \int_0^{L_p} C_p(x,t)\,\mathrm{d}x$ is the mass of drug per unit area and $M_0 = L_p C^0$. Evaluating the integration, the solution is readily seen to be (3.16).

**Appendix B**

In this appendix we provide an outline of the solution of the model presented in Section 3.3. The model presented in Section 3.2 is then shown to be a special case and we deduce the solution by taking the limit as $K_2 \to 0$. Consider the model of drug release by dissolution and diffusion as presented in Section 3.3:

$$\frac{\partial C_{\mathrm{f}}}{\partial t}(x,t) = D_P \frac{\partial^2 C_{\mathrm{f}}}{\partial x^2}(x,t) + K_1 C_{\mathrm{b}}(x,t) - K_2 C_{\mathrm{f}}(x,t), \quad 0 < x < L_{\mathrm{p}}, \quad t > 0, \tag{B.1}$$

$$\frac{\partial C_{\mathrm{b}}}{\partial t}(x,t) = -K_1 C_{\mathrm{b}}(x,t) + K_2 C_{\mathrm{f}}(x,t), \quad 0 < x < L_{\mathrm{p}}, \quad t > 0, \tag{B.2}$$

$$C_{\mathrm{b}}(x,0) = C_{\mathrm{b}}^0, \quad 0 \leqslant x \leqslant L_{\mathrm{p}}, \tag{B.3}$$

$$C_{\mathrm{f}}(x,0) = 0, \quad 0 \leqslant x \leqslant L_{\mathrm{p}}, \tag{B.4}$$

$$-D_{\mathrm{p}} \frac{\partial C_{\mathrm{f}}}{\partial x}(0,t) = 0, \quad t > 0, \tag{B.5}$$

$$C_{\mathrm{f}}(L_{\mathrm{p}},t) = 0, \quad t > 0. \tag{B.6}$$

Solving (B.2) subject to (B.3) provides

$$C_{\mathrm{b}}(x,t) = K_2 \int_0^t \exp\{-K_1(t-\tau)\} C_{\mathrm{f}}(x,\tau)\,\mathrm{d}\tau + C_{\mathrm{b}}^0 \exp\{-K_1 t\}. \tag{B.7}$$

Substitution of (B.7) into (B.1) and taking Laplace transforms we find that

$$\frac{\mathrm{d}^2 \bar{C}_{\mathrm{f}}}{\mathrm{d}x^2}(x,s) - \Gamma^2(s) = -\frac{K_1 C_{\mathrm{b}}^0}{D_{\mathrm{p}}(s+K_1)}, \tag{B.8}$$

where use has been made of (B.4), the overbar denotes the transformed variable, and

$$\Gamma^2(s) = \frac{s(s+K_2+K_1)}{D_{\mathrm{p}}(s+K_1)}. \tag{B.9}$$

Solving (B.8) subject to the conditions (B.5–B.6) yields

$$\bar{C}_{\mathrm{f}}(x,s) = \frac{K_1 C_{\mathrm{b}}^0}{s+K_2+K_1} \left( \frac{\cosh(\Gamma(s)L_{\mathrm{p}}) - \cosh(\Gamma(s)x)}{s\cosh(\Gamma(s)L_{\mathrm{p}})} \right). \tag{B.10}$$

We note that

$$L^{-1}\left[\frac{K_1 C_{\mathrm{b}}^0}{s+K_2+K_1}\right] = K_1 C_{\mathrm{b}}^0 \exp\{-(K_2+K_1)t\}. \tag{B.11}$$

Consider now

$$L^{-1}\left[\frac{\cosh(\Gamma(s)L_{\mathrm{p}}) - \cosh(\Gamma(s)x)}{s\cosh(\Gamma(s)L_{\mathrm{p}})}\right]. \tag{B.12}$$

By series expansion (or otherwise) it can be shown that expression (B.11) has no branch points. Furthermore, the apparent singularities at $s = 0$ and $s = -K_1$ are actually removable. Any other singularities must be such that

$$\cosh\left(\Gamma(s)L_{\mathrm{p}}\right)=0. \tag{B.13}$$

By writing the hyperbolic cosine in terms of exponentials it is readily seen that the roots of (B.13) must satisfy

$$\Gamma(s)L_{\mathrm{p}}=\mathrm{i}\pi\left(n-\tfrac{1}{2}\right), \quad n=\pm(1,2,3,\ldots). \tag{B.14}$$

Thus we have a countably infinite number of singularities. We proceed to invert (B.11) by use of the Residue Theorem. Substituting the expression for $\Gamma$ from (B.9) into (B.14) we see that the roots must satisfy the quadratic:

$$s^2+\left(K_2+K_1+\frac{D_{\mathrm{p}}}{4L_{\mathrm{p}}^2}\pi^2(2n-1)^2\right)s+\frac{D_{\mathrm{p}}}{4L_{\mathrm{p}}^2}\pi^2(2n-1)^2K_1=0. \tag{B.15}$$

The roots $s_{1_n}$ and $s_{2_n}$ are given by (3.30) from which we note that they all lie to the left of $s=0$. The residues are thus given by

$$\sum_{j=1}^{2}\sum_{n=1}^{\infty}\lim_{s\to s_{j_n}}\left\{\frac{s-s_{j_n}}{\cosh\left(\Gamma L_{\mathrm{p}}\right)}\right\}\lim_{s\to s_{j_n}}\left\{\frac{\left(\cosh\left(\Gamma L_{\mathrm{p}}\right)-\cosh\left(\Gamma x\right)\right)\exp\{st\}}{s}\right\}. \tag{B.16}$$

Making use of L'Hopital's rule, (B.16) becomes

$$\sum_{j=1}^{2}\sum_{n=1}^{\infty}\frac{(-1)^nD_{\mathrm{p}}(2n-1)\pi(s_{j_n}+K_1)^2\cos\left((2n-1)\pi x/2L_{\mathrm{p}}\right)\exp\{s_{j_n}t\}}{L_{\mathrm{p}}^2s_{j_n}(s_{j_n}^2+2K_1s_{j_n}+K_1(K_2+K_1))(K_2+K_1+s_{j_n})}. \tag{B.17}$$

Utilizing (B.11) and (B.17) along with convolution, the Laplace transform of (B.10) is

$$\begin{aligned}C_{\mathrm{f}}(x,t)=&\frac{K_1C_{\mathrm{b}}^0D_{\mathrm{p}}\pi}{L_{\mathrm{p}}^2}\int_0^t\exp\{-(K_2+K_1)(t-\tau)\}\\&\times\sum_{j=1}^{2}\sum_{n=1}^{\infty}\frac{(-1)^n(2n-1)(s_{j_n}+K_1)^2\cos\left((2n-1)\pi x/2L_{\mathrm{p}}\right)\exp\{s_{j_n}\tau\}}{s_{j_n}(s_{j_n}^2+2K_1s_{j_n}+K_1(K_2+K_1))(K_2+K_1+s_{j_n})}\,\mathrm{d}\tau.\end{aligned} \tag{B.18}$$

Evaluating the integral in (B.18) we obtain the solution (3.28). Furthermore, substitution of (3.28) into (B.7) provides the solution of bound drug concentration (3.29). The expression for the cumulative fraction of drug released is given by

$$M_{\mathrm{frac}}(t)=1-\frac{M(t)}{M_0}, \tag{B.19}$$

where $M_0=L_{\mathrm{p}}C_{\mathrm{b}}^0$ and

$$M(t)=\int_0^{L_{\mathrm{p}}}C_{\mathrm{b}}(x,t)\,\mathrm{d}x+\int_0^{L_{\mathrm{p}}}C_{\mathrm{f}}(x,t)\,\mathrm{d}x. \tag{B.20}$$

Evaluation of the integrals in (B.20) and substitution into (B.19) provides the solution of cumulative fraction of drug released as given by (3.31).

Now, it is readily seen that the model defined by (B.1–B.6) can be reduced to the model defined by (3.17–3.22) by letting $K_2 \to 0$. Taking this limit in (B.9),

$$\Gamma^2(s) \to \frac{s}{D_{\mathrm{p}}}, \tag{B.21}$$

so that, by (B.14),

$$s = -\frac{D_{\mathrm{p}}}{L_{\mathrm{p}}^2}\pi^2\left(n - \frac{1}{2}\right)^2, \quad n = \pm(1, 2, 3, \ldots) \tag{B.22}$$

are the singularities. In this limit,

$$\bar{C}_{\mathrm{f}}(x, s) = \frac{K_1 C_{\mathrm{b}}^0}{s + K_1}\left(\frac{\cosh\left(\sqrt{(s/D_{\mathrm{p}})}L_{\mathrm{p}}\right) - \cosh\left(\sqrt{(s/D_{\mathrm{p}})}x\right)}{s\cosh\left(\sqrt{(s/D_{\mathrm{p}})}L_{\mathrm{p}}\right)}\right). \tag{B.23}$$

Using the Residue Theorem as before, the solution is given by (3.24) and the corresponding expression for the cumulative fraction of mass released is given by (3.25).

## Appendix C

The following lemmas have to be used while solving Equation (5.1).

LEMMA C.1 Let $f(t)$ be any continuous function and let $\bar{f}(s) = L[f(t)]$ be its corresponding Laplace transform. Then if

$$\bar{f}(s) = \frac{1}{s}\exp\{-\sqrt{a(s+b)}\},$$

then

$$f(t) = \frac{1}{2}\left[\exp\{-\sqrt{ab}\}\,\mathrm{erfc}\left(-\sqrt{bt} + \frac{1}{2}\sqrt{\frac{a}{t}}\right) + \exp\{\sqrt{ab}\}\,\mathrm{erfc}\left(\sqrt{bt} + \frac{1}{2}\sqrt{\frac{a}{t}}\right)\right].$$

*Proof.* Note that one can write

$$\frac{1}{s}\exp\{-\sqrt{a(s+b)}\} = \left[\frac{-1}{\sqrt{s+b}+\sqrt{b}} + \frac{1}{\sqrt{s+b}-\sqrt{b}}\right]\frac{\exp\{-\sqrt{a(s+b)}\}}{2\sqrt{b}}. \tag{C.1}$$

Employing the Shifting Theorem, $L^{-1}[\bar{f}(s+b)] = \exp\{-bt\}L^{-1}[\bar{f}(s)] = \exp\{-bt\}f(t)$, results in

$$L^{-1}\left[\frac{1}{s}\exp\{-\sqrt{a(s+b)}\}\right]$$
$$= \frac{\exp\{-bt\}}{2\sqrt{b}}L^{-1}\left[\left\{\frac{1}{\sqrt{s}-\sqrt{b}} - \frac{1}{\sqrt{s}+\sqrt{b}}\right\}\exp\{-\sqrt{as}\}\right]$$

$$
\begin{aligned}
&= \frac{\exp\{-bt\}}{2\sqrt{b}} \\
&\times \left[ \begin{array}{l} \dfrac{1}{\sqrt{\pi t}} \exp\left\{-\dfrac{a}{4t}\right\} + \sqrt{b}\exp\{-\sqrt{ab}\}\exp\{bt\}\,\mathrm{erfc}\left(-\sqrt{bt} + \dfrac{1}{2}\sqrt{\dfrac{a}{t}}\right) \\ -\left(\dfrac{1}{\sqrt{\pi t}} \exp\left\{-\dfrac{a}{4t}\right\} - \sqrt{b}\exp\{\sqrt{ab}\}\exp\{bt\}\,\mathrm{erfc}\left(\sqrt{bt} + \dfrac{1}{2}\sqrt{\dfrac{a}{t}}\right)\right) \end{array} \right] \\
&= \frac{1}{2}\left[\exp\{-\sqrt{ab}\}\,\mathrm{erfc}\left(-\sqrt{bt} + \frac{1}{2}\sqrt{\frac{a}{t}}\right) + \exp\{\sqrt{ab}\}\,\mathrm{erfc}\left(\sqrt{bt} + \frac{1}{2}\sqrt{\frac{a}{t}}\right)\right]. \qquad \text{(C.2)}
\end{aligned}
$$

□

LEMMA C.2 Let $f(t)$ be any continuous function and let $\bar{f}(s) = L[f(t)]$ be its corresponding Laplace transform. Then if

$$\bar{f}(s) = \frac{1}{s}\exp\{-\sqrt{a(s+b)}\},$$

then

$$f(t) = \exp\{-bt\}\,\mathrm{erfc}\left(\frac{1}{2}\sqrt{\frac{a}{t}}\right) + b\int_0^t \exp\{-b\tau\}\,\mathrm{erfc}\left(\frac{1}{2}\sqrt{\frac{a}{\tau}}\right)\,\mathrm{d}\tau.$$

*Proof.* It is well established that $L^{-1}[\exp\{-\sqrt{as}\}/s)] = \mathrm{erfc}(\sqrt{(a/t)}/2)$. Also, from the first Shifting Theorem:

$$L^{-1}\left[\frac{1}{s+b}\exp\{-\sqrt{a(s+b)}\}\right] = \exp\{-bt\}L^{-1}\left[\frac{1}{s}\exp\{-\sqrt{as}\}\right]. \qquad \text{(C.3)}$$

Now

$$\frac{1}{s} = \left(1 + \frac{b}{s}\right)\frac{1}{s+b} \qquad \text{(C.4)}$$

so that

$$
\begin{aligned}
&L^{-1}\left[\frac{1}{s}\exp\{-\sqrt{a(s+b)}\}\right] \\
&\quad = L^{-1}\left[\left(1 + \frac{b}{s}\right)\frac{1}{s+b}\exp\{-\sqrt{a(s+b)}\}\right] \\
&\quad = \int_0^t [\delta(t-\tau) + b]\exp\{-b\tau\}\,\mathrm{erfc}\left(\frac{1}{2}\sqrt{\frac{a}{\tau}}\right)\mathrm{d}\tau \\
&\quad = \exp\{-bt\}\,\mathrm{erfc}\left(\frac{1}{2}\sqrt{\frac{a}{t}}\right) + b\int_0^t \exp\{-b\tau\}\,\mathrm{erfc}\left(\frac{1}{2}\sqrt{\frac{a}{\tau}}\right)\mathrm{d}\tau. \qquad \text{(C.5)}
\end{aligned}
$$

□

**Appendix D**

The two forms of solution, (5.4) and (5.5), can be shown to be equivalent. Consider firstly the results from Lemmas 1 and 2:

$$L^{-1}\left[\frac{1}{s}\exp\left\{\sqrt{a(s+b)}\right\}\right]$$
$$=\frac{1}{2}\left[\exp\{-\sqrt{ab}\}\,\mathrm{erfc}\left(-\sqrt{bt}+\frac{1}{2}\sqrt{\frac{a}{t}}\right)+\exp\{\sqrt{ab}\}\,\mathrm{erfc}\left(\sqrt{bt}+\frac{1}{2}\sqrt{\frac{a}{t}}\right)\right]. \tag{D.1}$$

$$L^{-1}\left[\frac{1}{s}\exp\{\sqrt{a(s+b)}\}\right]$$
$$=\exp\{-bt\}\,\mathrm{erfc}\left(\frac{1}{2}\sqrt{\frac{a}{t}}\right)+b\int_0^t\exp\{-b\tau\}\,\mathrm{erfc}\left(\frac{1}{2}\sqrt{\frac{a}{\tau}}\right)\mathrm{d}\tau. \tag{D.2}$$

Recall the definition of erfc$(z)$

$$\mathrm{erfc(z)}=\frac{2}{\sqrt{\pi}}\int_z^\infty\exp\{-u^2\}\,\mathrm{d}u. \tag{D.3}$$

Consider

$$\mathrm{erfc}\left(\frac{A}{k}+Bk\right)=\frac{2}{\sqrt{\pi}}\int_{A/k+Bk}^\infty\exp\{-u^2\}\,\mathrm{d}u. \tag{D.4}$$

Now, differentiating (D.4) with respect to $k$ provides (making use of Leibnitz rule)

$$\frac{\mathrm{d}}{\mathrm{d}k}\left[\mathrm{erfc}\left(\frac{A}{k}+Bk\right)\right]=-\frac{2}{\sqrt{\pi}}\exp\left\{-\left(\frac{A}{k}+Bk\right)^2\right\}\left(B-\frac{A}{k^2}\right)$$
$$=-\frac{2}{\sqrt{\pi}}\left(B-\frac{A}{k^2}\right)\exp\{-2AB\}\exp\left\{-\left(\frac{A}{k}\right)^2-(Bk)^2\right\}. \tag{D.5}$$

Similarly,

$$\frac{\mathrm{d}}{\mathrm{d}k}\left[\mathrm{erfc}\left(\frac{A}{k}-Bk\right)\right]=-\frac{2}{\sqrt{\pi}}\left(-B-\frac{A}{k^2}\right)\exp\{2AB\}\exp\left\{-\left(\frac{A}{k}\right)^2-(Bk)^2\right\}. \tag{D.6}$$

Thus one may write

$$\exp\{2AB\}\frac{\mathrm{d}}{\mathrm{d}k}\left[\mathrm{erfc}\left(\frac{A}{k}+Bk\right)\right]-\exp\{-2AB\}\frac{\mathrm{d}}{\mathrm{d}k}\left[\mathrm{erfc}\left(\frac{A}{k}-Bk\right)\right]$$
$$=-\frac{4B}{\sqrt{\pi}}\exp\left\{-\left(\frac{A}{k}\right)^2-(Bk)^2\right\}. \tag{D.7}$$

Noting that $-\mathrm{erfc}(z) = \mathrm{erfc}(-z)$, it then follows that

$$\begin{aligned}&\exp\{2AB\}\int_{(1/2)\sqrt{(a/t)}}^{\infty}\frac{\mathrm{d}}{\mathrm{d}k}\,\mathrm{erfc}\left(\frac{A}{k}+Bk\right)\mathrm{d}k\\&\quad+\exp\{-2AB\}\int_{(1/2)\sqrt{(a/t)}}^{\infty}\frac{\mathrm{d}}{\mathrm{d}k}\,\mathrm{erfc}\left(-\frac{A}{k}+Bk\right)\mathrm{d}k\\&=-\frac{4B}{\sqrt{\pi}}\int_{(1/2)\sqrt{(a/t)}}^{\infty}\exp\left\{-\left(\frac{A}{k}\right)^2-(Bk)^2\right\}\mathrm{d}k.\end{aligned}\tag{D.8}$$

Simplifying (D.8) provides

$$\begin{aligned}&\exp\{2AB\}\left[\mathrm{erfc}(\infty)-\mathrm{erfc}\left(\frac{2A\sqrt{t}}{\sqrt{a}}+\frac{B\sqrt{a}}{2\sqrt{t}}\right)\right]\\&\quad+\exp\{-2AB\}\left[\mathrm{erfc}(\infty)-\mathrm{erfc}\left(-\frac{2A\sqrt{t}}{\sqrt{a}}+\frac{B\sqrt{a}}{2\sqrt{t}}\right)\right]\\&=-\frac{4B}{\sqrt{\pi}}\int_{(1/2)\sqrt{(a/t)}}^{\infty}\exp\left\{-\left(\frac{A}{k}\right)^2-(Bk)^2\right\}\mathrm{d}k.\end{aligned}\tag{D.9}$$

Noting that $\mathrm{erfc}(\infty) = 0$, Equation (D.9) can be simplified further to give

$$\begin{aligned}&\frac{1}{2}\left[\exp\{2AB\}\,\mathrm{erfc}\left(\frac{2A\sqrt{t}}{\sqrt{a}}+\frac{B\sqrt{a}}{2\sqrt{t}}\right)+\exp\{-2AB\}\,\mathrm{erfc}\left(-\frac{2A\sqrt{t}}{\sqrt{a}}+\frac{B\sqrt{a}}{2\sqrt{t}}\right)\right]\\&=\frac{2B}{\sqrt{\pi}}\int_{(1/2)\sqrt{(a/t)}}^{\infty}\exp\left\{-\left(\frac{A}{k}\right)^2-(Bk)^2\right\}\mathrm{d}k.\end{aligned}\tag{D.10}$$

Making the substitution $B = 1$, $A = \sqrt{ab}/2$ in (D.10) results in the following expression:

$$\begin{aligned}&\frac{1}{2}\left[\exp\{-\sqrt{ab}\}\,\mathrm{erfc}\left(-\sqrt{bt}+\frac{1}{2}\sqrt{\frac{a}{t}}\right)+\exp\{\sqrt{ab}\}\,\mathrm{erfc}\left(\sqrt{bt}+\frac{1}{2}\sqrt{\frac{a}{t}}\right)\right]\\&=\frac{2}{\sqrt{\pi}}\int_{(1/2)\sqrt{(a/t)}}^{\infty}\exp\left\{-\frac{ab}{4k^2}-k^2\right\}\mathrm{d}k,\end{aligned}\tag{D.11}$$

whose right-hand side is a further characterization of the inverse Laplace transform:

$$L^{-1}\left[\frac{1}{s}\exp\{\sqrt{a(s+b)}\}\right]=\frac{2}{\sqrt{\pi}}\int_{(1/2)\sqrt{(a/t)}}^{\infty}\exp\left\{-\frac{ab}{4k^2}-k^2\right\}\mathrm{d}k.\tag{D.12}$$

Using (D.3), Equation (D.2) can be manipulated as follows:

$$\begin{aligned}
&\exp\{-bt\}\,\mathrm{erfc}\left(\frac{1}{2}\sqrt{\frac{a}{t}}\right) + b\int_0^t \exp\{-b\tau\}\,\mathrm{erfc}\left(\frac{1}{2}\sqrt{\frac{a}{\tau}}\right)\mathrm{d}\tau \\
&\quad = \exp\{-bt\}\,\mathrm{erfc}\left(\frac{1}{2}\sqrt{\frac{a}{t}}\right) + \frac{2b}{\sqrt{\pi}}\int_{(1/2)\sqrt{(a/t)}}^{\infty}\int_{a/4u^2}^{t} \exp\{-b\tau - u^2\}\,\mathrm{d}\tau\,\mathrm{d}u \\
&\quad = \exp\{-bt\}\left[\mathrm{erfc}\left(\frac{1}{2}\sqrt{\frac{a}{t}}\right) - \frac{2}{\sqrt{\pi}}\int_{(1/2)\sqrt{(a/t)}}^{\infty} \exp\{-u^2\}\,\mathrm{d}u\right] \\
&\qquad + \frac{2}{\sqrt{\pi}}\int_{(1/2)\sqrt{(a/t)}}^{\infty} \exp\left\{-\frac{ab}{4u^2} - u^2\right\}\mathrm{d}u \\
&\quad = \frac{2}{\sqrt{\pi}}\int_{(1/2)\sqrt{(a/t)}}^{\infty} \exp\left\{-\frac{ab}{4u^2} - u^2\right\}\mathrm{d}u. \qquad \text{(D.13)}
\end{aligned}$$

Making use of (D.11) and (D.13), one can write down the three equivalent forms:

$$\begin{aligned}
&L^{-1}\left[\frac{1}{s}\exp\{\sqrt{a(s+b)}\}\right] \\
&\quad = \frac{1}{2}\left[\exp\{-\sqrt{ab}\}\,\mathrm{erfc}\left(-\sqrt{bt} + \frac{1}{2}\sqrt{\frac{a}{t}}\right) + \exp\{\sqrt{ab}\}\,\mathrm{erfc}\left(\sqrt{bt} + \frac{1}{2}\sqrt{\frac{a}{t}}\right)\right] \\
&\quad = \exp\{-bt\}\,\mathrm{erfc}\left(\frac{1}{2}\sqrt{\frac{a}{t}}\right) + b\int_0^t \exp\{-b\tau\}\,\mathrm{erfc}\left(\frac{1}{2}\sqrt{\frac{a}{\tau}}\right)\mathrm{d}\tau \\
&\quad = \frac{2}{\sqrt{\pi}}\int_{(1/2)\sqrt{(a/t)}}^{\infty} \exp\left\{-\frac{ab}{4u^2} - u^2\right\}\mathrm{d}u. \qquad \text{(D.14)}
\end{aligned}$$

Thus, a third form of solution for drug concentration in the tissue is given by

$$C_{\mathrm{T}}(x,t) = \frac{2C^0}{\sqrt{\pi}}\exp\left\{\frac{v}{2D_{\mathrm{T}}}x\right\}\int_{(1/2)\sqrt{(a/t)}}^{\infty} \exp\left\{-\frac{ab}{4u^2} - u^2\right\}\mathrm{d}u. \qquad \text{(D.15)}$$
